# Rapid deployment valve system shortens operative times for aortic valve replacement through right anterior minithoracotomy

**DOI:** 10.1186/s13019-017-0598-0

**Published:** 2017-05-16

**Authors:** Constanze Bening, Khaled Hamouda, Mehmet Oezkur, Christoph Schimmer, Ina Schade, Armin Gorski, Ivan Aleksic, Rainer Leyh

**Affiliations:** 1 Department of Thoracic and Cardiovascular Surgery, University Hospital Wuerzburg, Wuerzburg, Germany; 2 Institute for Clinical and Biometry, University of Wuerzburg, Wuerzburg, Germany

**Keywords:** Aortic valve replacement, Minimally invasive surgery, Heart valve prosthesis biological rapid deployment aortic valve

## Abstract

**Background:**

There is growing evidence from the literature that right anterior minithoracotomy aortic valve replacement (RAT-AVR) improves clinical outcome. However, increased cross clamp time is the strongest argument for surgeons not performing RAT-AVR. Rapid deployment aortic valve systems have the potential to decrease cross-clamp time and ease this procedure. We assessed clinical outcome of rapid deployment and conventional valves through RAT.

**Methods:**

Sixty-eight patients (mean age 76 ± 6 years, 32% females) underwent RAT-AVR between 9/2013 and 7/2015. According to the valve type implanted the patients were divided into two groups. In 43 patients (R-group; mean age 74.1 ± 6.6 years) a rapid deployment valve system (Edwards Intuity, Edwards Lifesciences Corp; Irvine, Calif) and in 25 patients (C-group; mean age 74.2 ± 6.6 years) a conventional stented biological aortic valve was implanted.

**Results:**

Aortic cross-clamp (42.1 ± 12 min vs. 68.3 ± 20.3 min; *p* < 0.001) and bypass time (80.4 ± 39.3 min vs. 106.6 ± 23.2 min; *p* = 0.001) were shorter in the rapid deployment group (R-group). We observed no differences in clinical outcome. Postoperative gradients (R-group: max gradient, 14.3 ± 8 mmHg vs. 15.5 ± 5 mmHg (C-group), mean gradient, 9.2 ± 1.7 mmHg (R-group) vs. 9.1 ± 2.3 mmHg (C-group) revealed no differences. However, larger prostheses were implanted in C-group (25 mm; IQR 23–27 mm vs. 23 mm; IQR 21–25; *p* = 0.009).

**Conclusions:**

Our data suggest that the rapid deployment aortic valve system reduced cross clamp and bypass time in patients undergoing RAT-AVR with similar hemodynamics as with larger size stented prosthesis. However, larger studies and long-term follow-up are mandatory to confirm our findings.

## Background

Rapid deployment valve technologies have been introduced recently to simplify aortic valve replacement (AVR) [1–6]. Numerous publications in the last 3 years reflect the increasing interest of the cardiac surgical community [1–6]. Due to the growing number of transcatherter aortic valve replacements a rethinking of surgical strategies for surgical AVR is inevitable to sustain surgical AVR as the gold standard for the treatment of significant aortic valve stenosis. Although minimally invasive surgical techniques minimizing surgical trauma [7–15] may be one approach, only 24.6% of isolated surgical AVR in Germany are performed through a minimally invasive approach [16]. Basically, two different types of surgical minimally invasive aortic valve replacement techniques have been advocated, upper hemisternotomy AVR and right minithoracotomy AVR. The majority of minimally invasive surgical AVR are performed through an upper hemisternotomy. One reason for the reluctance to adopt minimal invasive technique might be a presumed longer aortic cross clamp and cardiopulmonary bypass time with negative effects for the patient. The data from recent publications clearly demonstrate the safety and effectiveness of rapid deployment valves, showing reduced aortic cross clamp and cardiopulmonary bypass time compared to standard stented biological valves [2, 17, 18]. Today only two rapid deployment valve systems are on the market, the Perceval valve (Sorin Group; Milan; Italy) on the one hand and the Edwards Intuity rapid deployment valve system (Edwards Lifescience Corp; Irvine, Calif; U.S.A.) on the other hand. However, only few studies published data regarding rapid deployment valves for RAT-AVR and none of them reported results with the Edwards Intuity Elite rapid deployment valve sytem [4, 5]. There is no data showing that the Edwards Elite valve system may result in shorter cross clamp and cardiopulmonary bypass times compared with conventional biological aortic valves, particularly through a right anterior minithoracotomy approach.

Therefore the aim of this study was to evaluate the impact of rapid deployment aortic valve replacement on procedural times and outcome in a cohort of patients undergoing right anterior minithoracotomy aortic valve replacement with the Edwards Intuity Elite rapid deployment valve system versus conventional biological valves.

## Methods

The ethics committee of the University Hospital Wuerzburg approved the present study. Due to the retrospective character of the study the ethics committee did not require written consent of the patients.

This was a retrospective study of prospectively collected data from consecutive patients scheduled for elective RAT-AVR at the Department of Thoracic and Cardiovascular Surgery in the University hospital Wuerzburg between 09/2013 and 07/2015.

Patients scheduled for elective surgical aortic valve replacement underwent 64 slice computed tomography (Somatom Definition ATS, Siemens, Germany) without contrast enhancement to evaluate the patient’s suitability for RAT-AVR. Basically, Glaubers criteria were used [7] to select patients for right anterior thoracotomy aortic valve replacement. The RD-valve system was the primary choice for AVR in these patients. Contraindications for implantation of an Edwards intuity RP valve system were a bicuspid aortic valve, non-calcified aortic valve pathology and an aortic valve annulus >27 mm in diameter. The later due to the maximum diameter of 27 mm provided by the company for this valve type. However, in some patients the adequately sized RD-valve was not available and a conventional stented biological valve was implanted instead.

From 09/2013 to 07/2015, 68 patients received a RAT-AVR for isolated aortic valve stenosis. According to valve type implanted the patients were divided into two groups. In one group (C-Group) a conventional stented biological valve was implanted. In the rapid deployment group (R-Group) the Edwards Intuity rapid deployment valve system was implanted. Pre-, perioperative and postoperative data were collected from patient records recorded. In addition to clinical variables, pre-and postoperative transthoracic echocardiographic parameters (mean-maximum valvular gradients and paravalvular leakage) were obtained from standardized echocardiography’s performed within the clinical routine. Patient-prosthesis mismatch (PPM) and an indexed effective orifice area (iEOA) < 0.65 cm^2^/m^2^ was considered as severe PPM and an iEOA <0.85 cm^2^/m^2^ as moderate PPM [19, 20]. Patient’s preoperative categorical and continuous data are depicted in Table 1.

**Table 1:** Patient’s characteristic

Variable	All pts.(*n* = 68)	C-Group-(*n* = 25)	R-Group(*n* = 43)	*p*-value
Age (y)	75.9 (SD 5.7)	74.2 (SD 6.6)	74.1 (SD 6.6)	0.058
BSA (m^2^)	1.89 (SD 0.17)	1.93 (SD 0.13)	1.86 (SD 0.18)	0.11
Female gender; n (%)	24 (35.3)	8 (32.0)	16 (37.7)	>0.5
COPD; n (%)	7 (10.3)	2 (8)	5 (11.6)	>0.5
DM; n (%)	26 (38.2)	5 (20)	21 (48.8)	0.022
Creatinine (mg/dl)	1.10 (0.86–1.4)	1.01 (0.7–1.5)	1.20 (1.0–1.4)	0.31
CKD-EPI (ml/min/1,73 m^2^)	64.5 (SD 21.2)	69.8 (SD 27.0)	61.5 (SD 16.5)	0.18
Chronic AF; n (%)	15 (22.1)	7 (28)	8 (18.6)	0.38
PAVD; n (%)	9 (13.2)	3 (12)	6 (14)	>0.5
Stroke; n (%)	4(5.9)	2(8.0)	2(4.7)	>0.5
Art.hypertension; n(%)	63(94)	21(87.5)	42(97.7)	0.13
Pul. hypertension; n (%)	24(35.3)	7(28.0)	17(39.5)	0.43
NYHA class III -IV; n (%)	40 (58.8)	14 (56)	26 (60.5)	>0.5
LVEF (%)	54 (SD 12.8)	54.2 (SD 14.0)	53.9 (SD 12)	0.25
EuroSCORE II (%)	5.3 (3.7–8.9)	5.0 (3.1–9.1)	5.7 (3.9–9.0)	0.29
Preoperative EOA, cm^2^	0.78 (SD 0.22)	0.75 (SD 0.25)	0.79 (SD 0.19)	>0.5
Preoperative mean aortic valve gradient, mmHG	49.6 (SD 11.6)	48.8 (SD 12.7)	50.1 (SD 10.9)	>0.5

Data presented as mean ± standard deviation (SD), median and IQR, or number of observations (n) with proportions (%)

*BSA* body surface area, *COPD* Chronic obstructive pulmonary disease, *DM* diabetes mellitus, *CKD-EPI* Chronic Kidney Disease Epidemiology Collaboration, *AF* atrial fibrillation, *NYHA* New York Heart Association Classification, *LVEF* left ventricular ejection fraction, *PAVD* peripheral arterial vascular disease; pulmonary hypertension: mean pulmonary pressure > 30 mmHG; *EOA* effective orifice area

### Surgical procedure

After induction of general anesthesia RAT-AVR was performed through a 5–6 cm skin incision placed above the third intercostal space in all patients, the fourth rib was separated from the sternum and carefully translocated caudally. The right mammary artery was severed and clipped in all patients. After dissection of mediastinal fat tissue the pericardium was opened longitudinally and multiple stay sutures were placed. A soft tissue retractor (Alexis Wound Protector, Applied Medical, USA), together with a small rib retractor (MRP-1, Fehling, Germany) was inserted to increase visualization of the surgical field. Direct ascending aortic cannulation with a flexible cannula (Easyflow Duo Cannulae, Sorin Group, Italy) was performed in 58 patients; in the remaining 10 patients peripheral arterial cannulation was performed. Indications for peripheral arterial cannulation were difficulties to visualize the area where to place the aortic cannula. Percutaneous venous cannulation (RAP femoral venous cannula, Sorin Group Italy) was used in all patients. Transesophageal echocardiogram was used in all patients to verify the correct position of the venous cannula in the vena cava superior and of the aortic cannula in the descending aorta, respectively.

Surgery was performed at moderate hypothermia (32 °C), standard myocardial protection was used. Before cross-clamping the aorta, a left atrial vent was placed in the right upper pulmonary vein in a usual fashion. After administration of cardioplegia a standard hockey stick incision towards the non coronary sinus extended below the sino tubular junction was performed to expose the native aortic valve and thorough decalcification of the aortic annulus was performed. At this point and after sizing the native aortic annulus the decision was made whether to implant a conventional stented biological valve or an Edwards Intuity rapid deployment valve. For implantation of a conventional standard valve pledgetted mattress sutures were placed in a standard fashion and the appropriate valve was positioned in a supraannular position. The following conventional valves were implanted: Trifecta (St, Jude Medical, St. Paul, MN, USA; *n* = 4), or Perimount (Edwards Lifesciences; *n* = 21).

For implantation of an Edwards Intuity rapid deployment valve a meticulous sizing technique has to be performed to omit the risk of malposition of the valve, since oversizing will result in valve luxation and undersizing in paravalvular leakage. Three guiding sutures were placed through the nadir of each sinus and through the corresponding part of the valve sewing ring. The valve system was lowered into the aortic annulus and proper seating of the valve was ensured. The guiding sutures were snared and the balloon catheter inflated for 10 s to deploy the stent frame. After deployment the delivery system and valve holder were removed carefully and the guiding sutures tied. In all patients the aortotomy was closed in a typical double layer fashion with a pledgetted suture starting at each corner of the aortotomy. After weaning from cardiopulmonary bypass standard transoesophegal echocardiographic examination of the implanted valve was performed to rule out any paravalvular leakage. Postoperative anticoagulation was in accordance with recent Guidelines for Management of Patients with Valvular Disease [21].

### Statistical analysis

Statistical analysis was performed using SPSS, version 23. Patient demographics are presented as mean ± standard deviation, medians and interquartile ranges and number of observations with proportions (%), as appropriate. Differences across group were assessed by t-Test, χ^2^-test/Fisher’s exact test, and Mann-Whitney-U-Test respectively. Two sided *p*-values < 0.05 were considered statistically significant.

## Results

Patient characteristics are shown in Table 1. Demographic data were comparable, the incidence of diabetes mellitus was higher in the R-group . Surgical data are depicted in Table 2. We observed shorter CPB-time (*p* = 0.001) and shorter aortic cross clamp-time (*p* < 0.001) for patients receiving Edwards Intuity rapid deployment valve (R-group). Aortic cross clamp time was reduced by 38% and CPB-time by 25%. A trend towards a shorter operative time was found (*p* = 0.071).

**Table 2:** Intraoperative characteristics

Variable	All pts.(*n* = 68)	C-Group-(*n* = 25)	R-Group(*n* = 43)	*p*-value
OP-time, min.	182 (167–207)	189 (169–219)	180 (152–197)	0.071
CPB-time, min.	90 (SD 36.4)	106.6 (SD 23.2)	80.4 (SD 39.3)	0.001
X-clamp time, min.	51.7 (SD 20.2)	68.3 (SD 20.3)	42.1 (SD 12.6)	<0.001
Conversion, n(%)	2 (2.9)	0 (0)	2 (4.6)	>0.5
Valve Diameter, mm	23 (21–25)	25 (23–27)	23 (21–25)	0.009

Data presented as mean ± standard deviation (SD), median and IQR, or number of observations (n) with proportions (%)

*OP* operation, *CPB* cardiopulmonary bypass

x-clamp, aortic cross clamp

Conversion rate for the entire patient cohort was 2.9% (2/68), with no differences between groups. One patient developed severe intraoperative bleeding due to an aortic root perforation. The patient required an aortic root replacement plus bypass surgery to the left coronary system due to laceration of the left main stem. The second patient became asystolic after central arterial cannulation, external chest compression was necessary, and the percutaneous femoral venous cannula was rapidly introduced to establish cardiopulmonary bypass. After uneventful AVR, severe hemorrhage developed. A full sternotomy revealed a laceration of the right ventricle caused by the percutaneous venous cannula as the bleeding source.

Biological valves with significantly larger diameter were implanted in the standard group (S-Group) (*p* = 0.009), this was due to fact that in the majority of these patients either a bicuspid aortic valve or a valve diameter >27 mm was detected during operation.

No differences in postoperative outcome were observed (Table 3). New onset atrial fibrillation had a low incidence in our patient cohort 8.8% (6/68). The same is true for atrioventricular block III° requiring pacemaker implantation (1.5%). However, three patients with temporary atrioventricular block III° were detected in the Edwards Intuity rapid deployment valve group (R-group), and one needed a permanent pacemaker implantation.

**Table 3:** Postoperative characteristics

Variable	All pts.(*n* = 68)	C-Group-(*n* = 25)	R-Group(*n* = 43)	*p*-value
Assisted ventilation (h)	11 (8–15)	12 (10–17)	11 (8–14)	0.14
Reoperation, n(%)	0 (0)	0 (0)	0 (0)	>0.5
Transfusion, n(%)	38 (57.4)	12 (48.0)	26 (60.5)	>0.5
RBC, units	0 (0–2)	0 (0–2)	0 (0–2)	>0.5
New–AF, n (%)	6 (8.8)	3 (12)	3 (7)	>0.5
Temp. AVB III°,n(%)	3 (4.4)	0 (0)	3 (7.0)	0.29
Perm. AVB III°,n(%)	1 (1.5)	0 (0)	1 (2.3)	>0.5
TND, n(%)	1(1.5)	1(4.0)	0 (0)	0.37
ICU stay, (d)	1(1–3)	1(1–3)	1(1–3)	>0.5
30d mortality, n(%)	2(2.9)	0(0)	2 (4.7)	>0.5

Data presented as mean ± standard deviation (SD), median and interquartile range (IQR), or number of observations (n) with proportions (%)

*RBC* red blood cell, *New-AF* new onset atrial fibrillation, *Temp AVB III°*, temporary atrioventricular block third degree; perm. *AVB III°* permanent atrioventricular block third degree, *TND* temporary neurological dysfunction, *ICU* intensive care unit

Overall 30 day mortality was 2.9% (2/68) for the entire patient cohort with no differences between groups. One patient who needed conversion to full sternotomy for aortic root replacement died on the 5^th^ postoperative day due to multiorgan failure, a second patient died on postoperative day 5 on the normal ward after an initial uneventful postoperative course. Since postoperative echocardiography on the same day had shown normal left and right ventricular function, low transprosthetic mean gradient and no paravalvular leakage it seems unlikely that the implanted valve caused this in-hospital death. However, we have no autopsy results on this patient.

Hemodynamic data showed no difference between groups for resting transvalvular mean (R group: 9.2 ± 1.7 mmHG, vs. 9.1 ± 2.3 mmHg (C-group), *p* > 0.5) and maximal gradients (R-group: 14.3 ± 8 mmHG, vs. 15.5 ± 5.0 mmHg, C-group, *p* = 0.30) respectively. In C-group significantly larger prostheses were implanted (Table 2), thus similar hemodynamics were found for the Rapid deployment as with larger stented conventional bioprosthesis. In neither group paravalvular leakage or PPM were detected.

## Discussion

We demonstrated a reduced aortic cross clamp-time and CPB time with the Edwards’s Intuity rapid deployment valve system compared with conventional stented biological valves for AVR through right anterior thoracotomy. To the best of our knowledge there are no data about aortic cross clamp and CPB time for the Edwards Intuity valve system in right minithoracotomy AVR. The mean aortic cross clamp time for the Edwards Intuity rapid deployment valve system in our study with 42.1 ± 12.6 min and CPB time of 80.4 ± 39.3 min are comparable to isolated AVR with the same valve type in the setting of either hemi- or full- sternotomy AVR [3, 11]. Only Schlömicher and coworker demonstrated a markedly reduced aortic cross clamp time for the Edwards Intuity rapid deployment valve (26 ± 7 min.) in hemisternotomy AVR [2]. A randomized comparison between patients undergoing minimally invasive AVR with rapid deployment valve systems and standard stented biological valves has not been reported yet. The only randomized study compared minimally invasive rapid deployment versus conventional full sternotomy aortic valve replacement; in this study a significantly reduced aortic cross clamp time was observed for rapid deployment AVR but no difference in clinical outcome [3].

Advantages for right mini-thoracotomy AVR compared to full-sternotomy AVR have been published [9, 11, 22]. The benefits included shorter mechanical ventilation time, shorter intensive care unit stay and hospital length of stay, lower incidence of blood transfusion and postoperative atrial fibrillation. However, consistent disadvantages were increased aortic cross-clamp and cardiopulmonary bypass time. A recent Bayesian network meta-analysis comparing ministernotomy or minithoracotomy for minimalinvasive AVR revealed longer aortic cross clamp and CPB time for minithoracotomy AVR with similar post-operative outcomes compared to ministernotomy AVR [14]. It has been shown that aortic crossclamp time > 60 min and prolonged cardiopulmonary bypass time are independent predictors of mortality and morbidity in low- and high-risk cardiac patients [23, 24]. In minithoracotomy AVR exposure and implantation of the prosthesis is more challenging than for full sternotomy or hemisternotomy AVR, which could be the reason for longer aortic cross clamp and CPB time. Therefore right minithoracotomy AVR is generally considered a challenging approach reserved for very experienced surgeons. New technologies, like sutureless or rapid deployment valves facilitate right minithoracotomy AVR due to a faster and easier implantation technique. Glauber and coworkers were able to reduce aortic cross clamp for minithoracotomy AVR by 28 min when using a sutureless Perceval S prosthesis (Sorin Biomedica) [4, 9]. In this study we reduced the aortic cross clamp time and cardiopulmonary bypass time by 26 min each using the Edwards Intuity rapid deployment valve.

Valve hemodynamics at discharge showed equally good results for all valve types with low mean pressure gradients at. However, in the patients with standard stented bioprosthesis larger valves were implanted. Therefore, it can be assumed that there might be a hemodynamic advantage for the Edwards intuity rapid deployment compared to conventional stented valves. The specific design of the rapid deployment valve could explain this result. Basically, the Edwards intuity valve is a stented bovine pericardial bioprosthesis attached to a balloon expandable, cloth covered skirt frame at the inflow part of the valve. After inflation of the balloon the skirt frame will expand and create a reversed funnel thereby widening the left ventricular outflow tract. This could result in less turbulence with subsequently better hemodynamic. Our results are supported by Borger and coworkers who showed lower mean gradients after 3 months and lower incidence of PPM for the Edwards Intuity valve compared to standard stented valves, although the implanted valve size revealed no differences [3].

Overall complication rate in this study was remarkably low. Neither PPM nor paravalvular leakage was detected in this series independent from the implanted valve type. The incidence of pacemaker implantation for new atrioventricuar block III° was 1.5% (1/68) for the entire patient cohort and 2.3% (1/43) in the rapid deployment group (R-group). It is noteworthy that after implantation of the Edwards intuity valve a 7%(3/43) incidence of temporary atrioventricuar block III° occurred but did notin the conventional group. Whether balloons expansion of the subvalvular skirt frame has any negative impact on the conduction system has not been evaluated in detail yet.

Low complication rates, good hemodynamics and the non-sternal splitting minimally invasive approach of minithoracotomy AVR in combination with new rapid deployment or sutureless valves AVR justifies that this technique should be added to the armamentarium in the treatment of aortic valve stenosis. Furthermore, there are data indicating that the combination of rapid deployment or sutureless valves with minimally invasive AVR approaches show better clinical results in high risk patients compared with transapical transcatheter aortic valve implantation [25, 26] In view of the increasing competition with transcatheter approaches for patients with an intermediate and high risk profile this technique deserves comparison with transfemoral transcatheter aortic valve implantation.

### Limitation of the study

Several facts could have influenced our results.

1. The fact that this is not a randomized study and the number of patients evaluated is relatively small should lead to a cautious interpretation of these data. However, the reduction in aortic cross clamp time and CPB time was striking and highly significant. The effectiveness and time saving effect of the Edwards Intuity rapid deployment valve system implanted through a right anterior mini-thoracotomy have been proven.
2. A selection bias cannot be ruled out entirely. Contraindication for placing the Edwards RD-valve system was a bicuspid aortic valve, and an annulus size > 27 mm. Furthermore, lack of availability ofadequately sized valves precluded implantation of RD-valve system in several patients, and this could have influenced our result. However, difficulties in placing interrupted mattress sutures in the aortic annulus are not influenced by the size of the annulus, but rather by difficulties in proper exposition of the native annulus through a right anterior mini-thoracotomy. Therefore we believe that our results reflect daily surgical practice when performing right anterior mini-thoracotomy AVR.

## Conclusion

Our preliminary data indicate that the implantation of Edwards Intuity RD-valve system via a right mini-thoracotomy is safe and reproducible. This valve system reduced aortic cross clamp and CPB time compared with conventional stented biological valves thereby supporting further spreading of this innovative surgical approach for isolated surgical AVR. These results could accelerate a rethinking in surgical strategies for non-sternal surgical AVR. For final judgment of this RD-valve system for RAT-AVR larger studies comparing different RD-valve systems and conventional stented biological valves are mandatory. Furthermore, long-term follow up is necessary to evaluate the longevity of the valve and to prove the good hemodynamics in the long run.

## Abbreviations

AVR: Aortic valve replacement
CPB: Cardiopulmonary bypass
iEOA: Indexed effective orifice area
PPM: Patient prosthesis mismatch
RAT: Right anterior thoracotomy
RAT-AVR: Right anterior thoracotomy-aortic valve replacement
RD: Rapid deployment
